# Accelerated Hyperfractionated Radiotherapy versus Conventional Fractionation Radiotherapy for Head and Neck Cancer: A Meta-Analysis of Randomized Controlled Trials

**DOI:** 10.1155/2019/7634746

**Published:** 2019-11-28

**Authors:** Bo Zhu, Changgui Kou, Wei Bai, Weiying Yu, Lili Zhang, Xiao Yu, Wen Xu, Huanhuan Wang, Ying Xin, Xin Jiang

**Affiliations:** ^1^Department of Epidemiology and Biostatistics, School of Public Health, Jilin University, Changchun, Jilin Province, China; ^2^Department of Radiation Oncology, The First Hospital of Jilin University, Changchun, Jilin Province, China; ^3^Department of Social Medicine and Health Management, School of Public Health, Jilin University, Changchun, Jilin Province, China; ^4^Department of Pathology, School of Basic Medicine, Jilin University, Changchun, Jilin Province, China

## Abstract

**Objective:**

The benefits of accelerated hyperfractionated radiotherapy (HART) and conventional fractionation radiotherapy (CFRT) in the treatment of head and neck cancer (HNC) remain controversial. In this study, we analyzed the therapeutic effects of these two treatment regimens to explore whether HART can improve the overall survival (OS) rate and locoregional control (LRC) rate in patients with HNC.

**Methods:**

The PubMed, EMBASE, and Cochrane Central Register of Controlled Trials (CENTRAL) databases were searched for eligible studies. The OS rate and LRC rate were considered as the efficacy outcomes. *I*^2^ was used to test the heterogeneity among studies with a cutoff value of 50%. Potential publication bias was assessed by funnel plots and Egger's test. We also performed a sensitivity analysis to assess the stability of the results. In this meta-analysis, all analyses were performed using R 3.5.3 software.

**Results:**

Twelve qualified articles including a total of 2,935 patients were identified. HART had a significant beneficial effect on OS rate (HR = 0.80, 95% CI: 0.65–0.98). Compared with CFRT, HART demonstrated a significantly higher LRC rate (HR = 0.82, 95% CI: 0.71–0.96).

**Conclusion:**

Our meta-analysis showed that HART can significantly improve OS and LRC compared with CFRT in patients with HNC.

## 1. Introduction

Head and neck cancer (HNC) is one of the most common types of cancer, with estimated more than 600,000 new cases (including cancer of the oral cavity, oropharynx, hypopharynx, and larynx) each year and more than 300,000 deaths all over the world [1, 2]. Today, HNC has become the main social burden both in developing and developed countries [3]. Approximately 40% of patients have developed locally advanced disease at the time of diagnosis. Surgery, radiation therapy, chemotherapy, and targeted therapy or different combinations of these therapies have been the primary treatments in the past few decades. The most common treatment regimen is conventional fractionation radiotherapy (CFRT) with a dose of 2.0 Gy/fraction/day, 5 days a week for 6-7 weeks. Despite the use of various treatment modalities, the prognosis of patients with locally advanced HNC is still poor, with a 5-year overall survival (OS) rate of 30∼35% [4].

Since the 1980s, unconventional fractionation therapy methods have been developed, and new treatment options for HNC have been tested several times [5]. The differences between various types of unconventional fractionation radiation depend on their dose of radiation, the number of radiation session, and the total duration of radiotherapy. Accelerated hyperfractionated radiotherapy (HART) is a common treatment among the unconventional fractionation therapy options [6]. The HART plan has more daily radiotherapy times and treatment doses than the CFRT plan does [7]. In some randomized controlled trials, the frequency of treatment per day for HART was more than that for CFRT, the average dose per fractionation was greater than that of CFRT, and the average total time was less than that of CFRT. The choice of HART treatment may not only reduce tumor regeneration by shortening the overall treatment time, which may improve local tumor control rates but also increase economic efficiency by reducing the treatment time [8]. Although there were several high-quality papers [9, 10] which showed that HART was superior to CFRT, some studies [11, 12] have found that HART was not better than CFRT. Therefore, we performed a meta-analysis to investigate the prognostic effect of HART and CFRT for HNC. The main purpose was to study the effect of HART and CFRT on the OS rate and locoregional control (LRC) rate to provide guidance for a reasonable clinical practice.

## 2. Materials and Methods

### 2.1. Search Strategy and Study Selection

This systematic review was conducted under the recommendations of the Preferred Reporting Items for Systematic Reviews and Meta-Analyses (PRISMA) statement [13]. This meta-analysis has been registered in the International Prospective Register of Systematic Reviews (PROSPERO), and the registration number is CRD42019121792. We thoroughly searched PubMed, EMBASE, and Cochrane Central Register of Controlled Trials (CENTRAL) from inception to 31 December 2018. The search terms were a combination of keywords and free words, and the following keywords were used: “head and neck cancer,” “hyperfraction^*∗*^,” “accelerated fractionation^*∗*^,” “conventional fractionation,” and “randomized controlled trial.” According to different databases, the search strategy was adjusted accordingly, and all search strategies were determined by multiple preretrieval tests combining the keywords and free words. We required the literature to be in English, and the research was limited to human subjects.

### 2.2. Inclusion and Exclusion Criteria

1. Before treatment, patients diagnosed with HNC (including nasopharyngeal, oral cavity, oropharyngeal, hypopharyngeal, esophageal, and laryngeal carcinomas) were included.
2. Patients treated with HART (including late-course HART, split-course HART, continuous HART, or other types) and CFRT were included.
3. OS and LRC were used to evaluate treatment efficacy. Studies providing sufficient information to estimate the hazard ratio (HR) and 95% confidence interval (CI) for the OS or LRC rates were included.
4. Randomized controlled trials were included in the analysis. Studies with no randomization, case reports, conference papers or abstracts, letters, review studies, and studies with no data available were excluded.

#### 2.2.1. Data Extraction and Quality Assessment

Data extraction and quality assessment were performed independently by the two review authors, and discrepancies were identified and resolved through discussion (with a third author when necessary). HR was an appropriate clinically relevant measure of overall effect [14]. The following information was extracted: first author's name, year of publication, gender ratio, total number of patients, number of patients in the CFRT and HART groups, median follow-up time, Karnofsky performance score (KPS), tumor site, cancer stage or T and N classifications, allocated treatment schedule, and HR and 95% CI for OS rate and/or LRC rate. If the literature did not directly provide original data or data on the HR and 95% CI, then we extracted the data from the survival curve and estimated them using Engauge Digitizer version 4.3 software. We evaluated the quality assessment with Review Manager (version 5.3; the Cochrane Collaboration, Oxford, UK), including the following items: generation of a randomization sequence, allocation concealment, blinding, incomplete outcome data, selective reporting, and other bias.

### 2.3. Statistical Analysis

In this meta-analysis, all of our analyses were performed using the “meta” package in R software version 3.5.3. Before calculating the combined HR and 95% CI, we analyzed the heterogeneity of the studies. *I*^2^ was adapted to evaluate the heterogeneity among studies. If there was statistical heterogeneity (*I*^2^ > 50%), we used the random effects model for analysis (DerSimonian and Laird methods); otherwise, the heterogeneity was evaluated by the fixed effects model (Mantel and Haenszel methods). A significantly lower HR (HR < 1) indicated that one treatment may be more effective than the other, with 95% CI in this range not including 1. Potential publication bias was assessed by funnel plots and Egger's test, and *P* values higher than 0.05 indicated that there was no publication bias. We also performed a sensitivity analysis to assess the stability of the results.

## 3. Results

### 3.1. Literature Search

**Figure 1:**
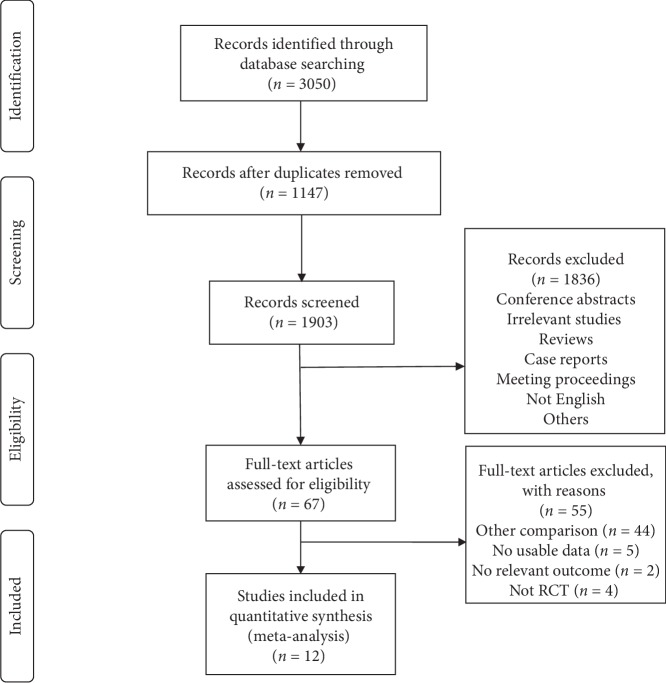
Flow diagram of the retrieved studies.

In this study, 3,050 articles were identified by the search strategy, and 1,903 articles were assessed after the duplicates were excluded. After screening the title and abstract and reading full texts, 55 of the 67 articles were subsequently excluded from the meta-analysis: 44 studies were related to other comparisons, 5 studies lacked usable data, 2 studies did not report relevant outcome, and 4 studies were not RCT. Finally, a total of 12 articles were included [7, 9–12, 15–21]. The PRISMA research flowchart is shown in Figure 1.

### 3.2. Study Characteristics and Quality Assessment of the Included Studies

**Figure 2:**
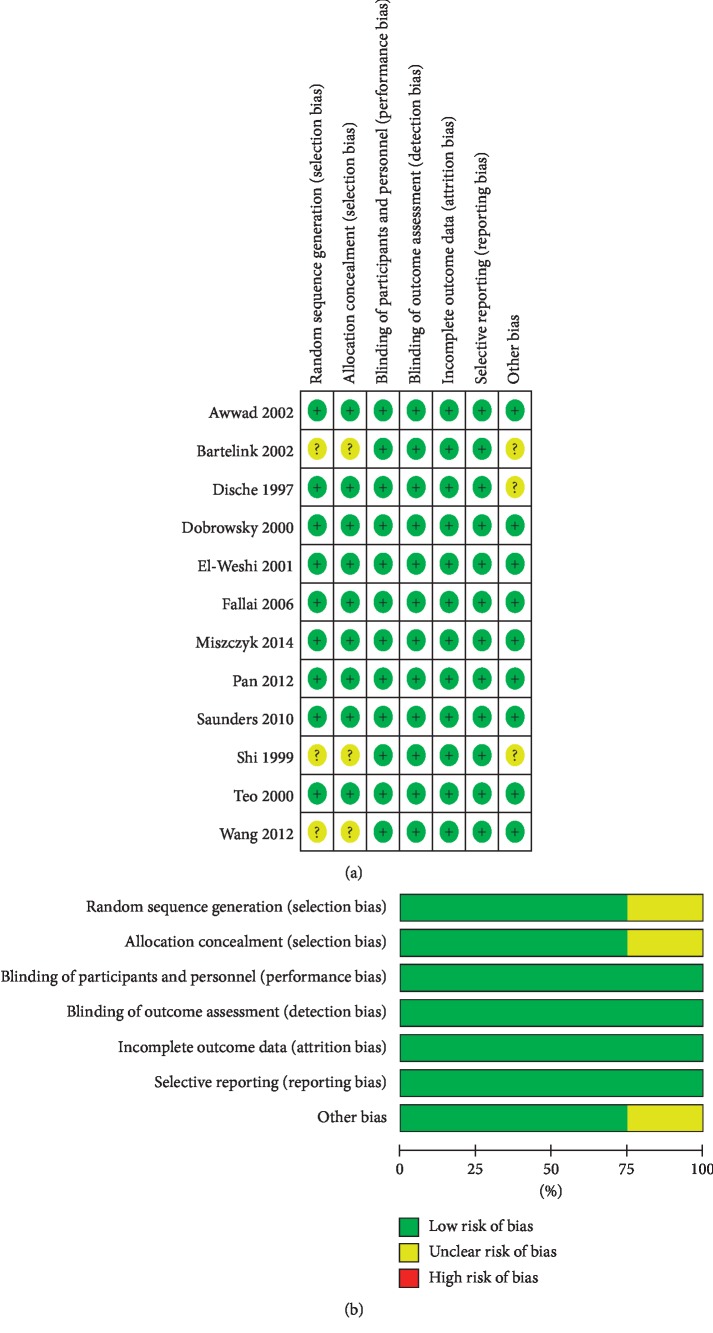
Assessment of study quality. (a) Risk of bias summary. (b) Risk of bias graph.

This meta-analysis included 2,935 patients. The study characteristics, including the first author's last name, publication year, number of patients, sex ratio, and other characteristics, are summarized in Table 1. The quality assessment of the 12 included studies is presented in Figure 2. The results showed that the quality of the included studies was generally high.

### 3.3. Outcomes

**Figure 3:**
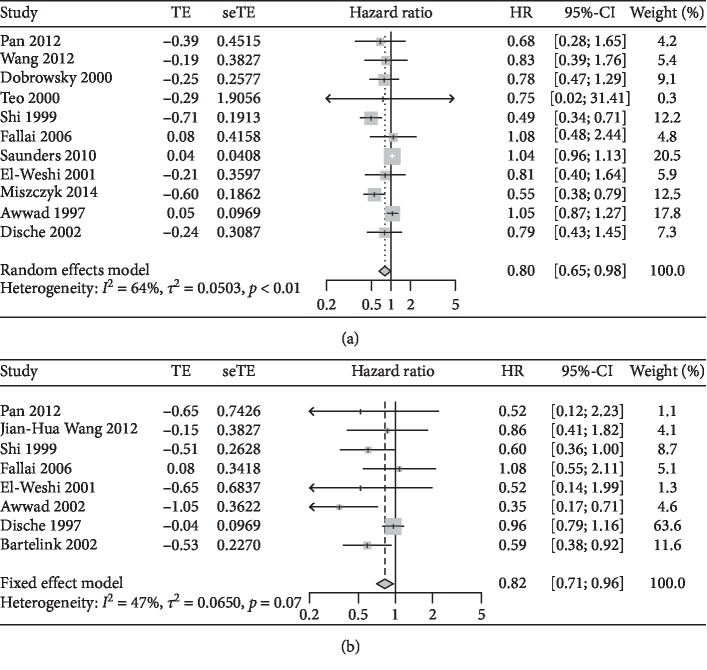
Comparison of the treatment efficiency between HART and CFRT. (a) Overall survival (OS) rate. (b) Locoregional control (LRC) rate.

There was evidence of heterogeneity between the two arms of the OS rate; thus, the random effects model was chosen for the meta-analysis (*I*^2^ = 64.0%); HART was associated with a significant benefit on the OS rate compared with CFRT (HR = 0.80, 95% CI: 0.65–0.98) (Figure 3(a)). From the results, there was no evidence of heterogeneity between the two arms of the LRC rate; thus, a fixed effects model was chosen for the meta-analysis (*I*^2^ = 47.0%); when compared with CFRT, HART demonstrated a significantly higher LRC rate (HR = 0.82, 95% CI: 0.71–0.96) (Figure 3(b)).

### 3.4. Sensitivity Analysis and Publication Bias

**Figure 4:**
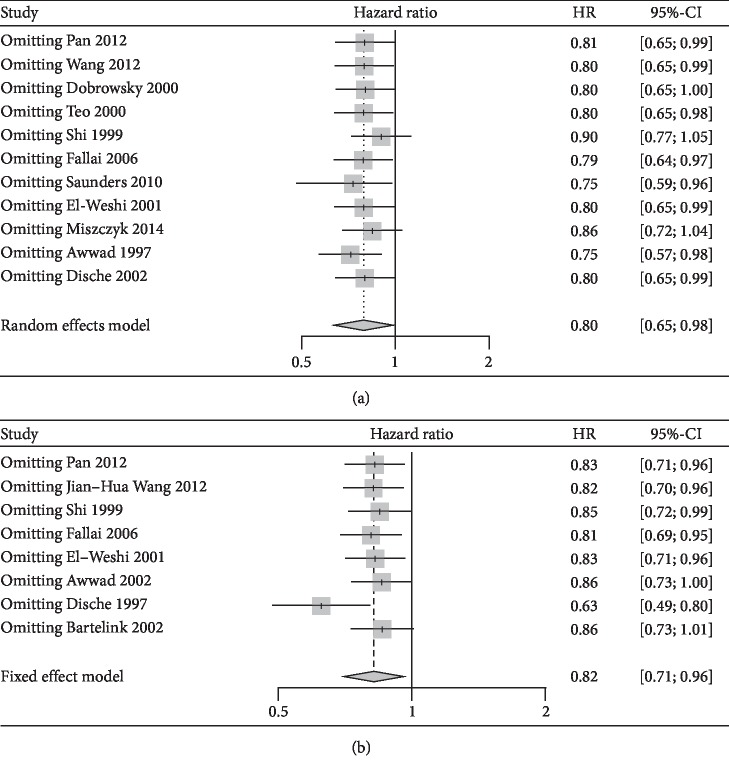
Sensitivity analyses of treatment efficiency between HART and CFRT. (a) Overall survival (OS) rate. (b) Locoregional control (LRC) rate.

**Figure 5:**
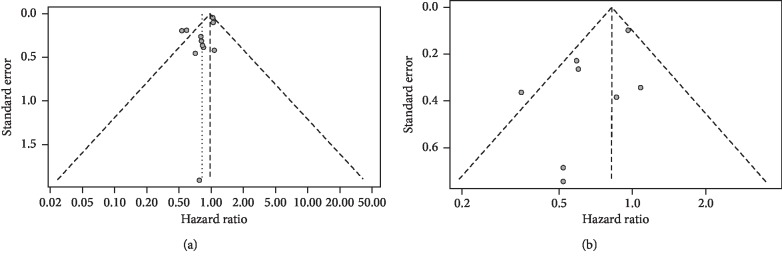
Funnel plots of treatment efficiency between HART and CFRT. (a) Overall survival (OS) rate. (b) Locoregional control (LRC) rate.

A sensitivity analysis was used to assess the stability of the combined results. We found that the results for OS rate (Figure 4(a)) and LRC rate (Figure 4(b)) may not be stable. The funnel plots of OS rate and LRC rate are shown in Figure 5. The funnel plots were symmetrically distributed, and Egger's test was used to assess publication bias (OS: *P* value = 0.058, LRC: *P* value = 0.098). There was no publication bias found using these indicators.

## 4. Discussion

HNC is a high-mortality cancer with a mortality rate of approximately 50% [22]. Surgery is one of the standard treatments for patients with HNC, but the treatment effect and prognosis remain poor [23]. The main cause of failure of locally advanced HNC surgery is local recurrence caused by regrowth of tumor cell residues [14]. Radiotherapy has become an important nonsurgical treatment for different stages of HNC, especially advanced lesions that usually recur in local areas [24]. Most clinical practices also suggest that the main cause of failure by CFRT for malignant tumors is local recurrence. Tumor repopulation caused by accelerated proliferation of tumor surviving cells during radiotherapy is considered to be a major factor in treatment failure [25]. Therefore, the control of tumor stem cells in the head and neck becomes the key to improving patient survival and local control rates. The accelerated proliferation of tumors is closely related to the potential doubling time. Cellular dynamics studies show that many tumors originating in the head and neck have a short potential tumor doubling time of less than 5 days [25–27]. The time used in the CFRT protocol may be detrimental to the treatment of HNC. A treatment plan that shortens the total treatment time is beneficial to improving the patient's LRC rate. In the past few decades of research and clinical applications, unconventional fractionation radiotherapy has been applied and developed [28, 29].

In other cancers, a large number of studies have shown that HART is superior to CFRT in the effect of treatment on prognosis; thus, we explored the efficacy of HART and CFRT in the treatment of HNC. In a meta-analysis of esophageal cancer [30], it was reported that HART improved the response rate, 1-, 3-, and 5-year OS rates, and the 1-, 3-, and 5-year local control rates compared with CFRT. A meta-analysis of lung cancer treatments also showed that the HART regimen improved the OS rate compared with the CFRT regimen (HR = 0.88; 95% CI: 0.80–0.97). In this study, we analyzed 12 papers involving a total of 2,935 patients with HNC. The study mainly analyzed the effectiveness of HART in the treatment of HNC compared with the effectiveness of CFRT. The OS rate and LRC rate were chosen as the primary outcomes of the study analysis. Our meta-analysis showed that compared to CFRT, HART had a certain benefit in the treatment of patients with HNC, and the OS rate (HR = 0.80, 95% CI: 0.65–0.98) and LRC rate (HR = 0.82, 95% CI: 0.71–0.96) were improved. This finding is consistent with other researchers' conclusions in other cancers. The assessment of study quality showed that the included studies were of high quality. The theoretical advantage of the HART plan is the possibility of controlling the chance of tumor cell proliferation by shortening the total treatment time. At the same time, increasing the daily dose of radiotherapy is also an important way to overcome the reproduction of tumor cells. A meta-analysis of HNC showed that altered fractionation radiotherapy (hyperfractionated, moderately accelerated, and very accelerated) was superior to conventional fractionation radiotherapy, and hyperfractionation was the better altered fractionated schedule when radiotherapy alone was used, which might be related to the increase in the absolute dose provided by hyperfractionation [31]. It can thus be explained that the HART regimen increases the probability of tumor control compared with the CFRT regimen. Furthermore, changes in total treatment time and total radiation dose may also have an impact on the results of acute and late toxicity in HNC patients. However, due to data limitations, toxicity outcomes were not analyzed in this meta-analysis. There are several limitations in this meta-analysis. First, most of the included references do not directly provide indicators of the results. There may be bias in extracting analytical data from survival curves. Second, we have conducted a subgroup analysis, but the results showed that there was no significant difference between different indicators (for example, the different sites of the cancer). At the same time, the sensitivity analysis results show that the OS and LRC rates may not be stable. Third, we combined continuous acceleration hyperfractionated treatment and split or late accelerated hyperfractionated treatment into the group described as the HART treatment plan, which may have a certain impact on our research results. However, our results still provide some useful information, and we need more experiments in the future to indicate whether the treatment effect of HART is better than that of CFRT.

## 5. Conclusions

In conclusion, our meta-analysis showed that HART was superior to CFRT in patients with HNC and that HART can improve patient OS and LRC rates compared with CFRT.

## Figures and Tables

**Table 1:** Study characteristics of the included studies.

No	Reference	Country	*N* patients	Gender (M/F)	Age	KPS	Tumor site	Stage	Follow-up (median or mean)	Arm	Dose/fraction (Gy)	Total dose (Gy)
1	Pan et al. [15]	China	200	150/50	49 (18–70)	≥70	Nasopharynx	I–IV	6.9 years	HART	1.2–1.5	78
										CFRT	2	70
2	Wang et al. [16]	China	98	63/35	65 (55–74)	≥70	Esophageal	—	45 (36–58) months	HART	1.5	64 (61–67)
										CFRT	2	64 (60–68)
3	Dobrowsky and Naude [9]	Ireland	159	139/22	34–77	90–100	Oral cavity Oropharynx Hypopharynx Larynx	T1–T4 N0–N3	48 months	HART	1.65–2.5	55.3
										CFRT	2	70
4	Teo et al. [17]	China	159	122/37	—	—	Nasopharynx	II–IV	59.2 months	HART	1.5	22.4
										CFRT	2.5	20
5	Shi et al. [18]	China	85	50/35	55.6	>70	Esophagus	—	5 years	HART	1.5–1.8	68.4
										CFRT	1.8	68.4
6	Fallai et al. [11]	Italy	128	112/16	—	≥70	Oropharynx	III-IV	8.35 (4.8–10.2) years	HART	1.6	64–67.2
										CFRT	—	66–70
7	Saunders et al. [19]	United Kingdom	918	—	—	—	—	T2–T4 N0-N1 M0	≤6 years	HART	1.5	54
										CFRT	2	66
8	El-Weshi et al. [20]	Egypt	50	40/10	39.9 (18–63)	—	Nasopharynx	III-IV	55 (4–120) months	HART	1.6	72
										CFRT	2	72
9	Miszczyk et al. [21]	Poland	101	78/23	57 (42–73)	—	Excluding nasopharynx	T2N3 T3N03 T4N0-N3	—	HART	1.6	64
										CFRT	2	50
10	Awwad et al. [12]	Egypt	70	56/14	50 (25–65)	—	Oral cavity Hypopharynx Larynx	T2–T4	—	HART	1.4	46.2
										CFRT	2	60
11	Dische et al. [7]	United Kingdom	918	687/231	—	—	Nasal sinus Nasopharynx Oral cavity Oropharynx Hypopharynx Larynx	T1–T4	N0–N3	HART	1.5	54
										CFRT	2	66
12	Bartelink et al. [10]	Netherlands	49	38/11	—	—	Oral cavity Oropharynx Larynx Hypopharynx	T2–T4	—	HART	1.6	72
										CFRT	2	70

Notes: “–,” not mentioned. CFRT, conventional fractionation radiotherapy; HART, accelerated hyperfractionated radiotherapy.

## Data Availability

The data used to support the findings of our study have been deposited in PubMed. All 12 articles included in our meta-analysis can be found in PubMed; the PMID are 22134512, 21858589, 11121628, 11054514, 10386713, 16683383, 20394851, 11669328, 25027170, 11870530, 9288840, and 11916549, respectively.

## Supplementary Materials

Supplementary Materials The supplementary material we submitted is the PRISMA Checklist, which contains a list of 27 items as supporting information for our article.
